# Suprascapular nerve block is a clinically attractive alternative to interscalene nerve block during arthroscopic shoulder surgery: a meta-analysis of randomized controlled trials

**DOI:** 10.1186/s13018-021-02515-1

**Published:** 2021-06-11

**Authors:** Changjiao Sun, Xiaolin Ji, Xiaofei Zhang, Qi Ma, Peng Yu, Xu Cai, Huadong Yang

**Affiliations:** 1 Department of Orthopedic, Beijing Tsinghua Changgung Hospital, School of Clinical Medicine, Tsinghua University, No. 168 Litang Road, Dongxiaokou Town, Changping District, Beijing, 102218 China; 2 Department of Anesthesia, Beijing Tsinghua Changgung Hospital, School of Clinical Medicine, Tsinghua University, No. 168 Litang Road, Dongxiaokou Town, Changping District, Beijing, 102218 China; 3 Department of Clinical Epidemiology and Biostatistics, Beijing Tsinghua Changgung Hospital, School of Clinical Medicine, Tsinghua University, No. 168 Litang Road, Dongxiaokou Town, Changping District, Beijing, 102218 China; 4 Department of Orthopedic, Wuhan University of Science and Technology Hospital, Qingling Street, Hongshan District, Wuhan, 102218 China

**Keywords:** Nerve block, Regional, Suprascapular, Interscalene, Shoulder, Arthroscopy

## Abstract

**Background:**

The interscalene brachial plexus block (ISB) is a commonly used nerve block technique for postoperative analgesia in patients undergoing shoulder arthroscopy surgery; however, it is associated with potentially serious complications. The use of suprascapular nerve block (SSNB) has been described as an alternative strategy with fewer reported side effects for shoulder arthroscopy. This review aimed to compare the impact of SSNB and ISB during shoulder arthroscopy surgery.

**Methods:**

A meta-analysis was conducted to identify relevant randomized controlled trials involving SSNB and ISB during shoulder arthroscopy surgery. Web of Science, PubMed, Embase, Cochrane Controlled Trials Register, Cochrane Library, Highwire, CNKI, and Wanfang database were searched from 2010 through March 2021.

**Results:**

We identified 1255 patients assessed in 17 randomized controlled trials. Compared with the ISB group, the SSNB group had higher VAS at rest in PACU (*P* = 0.003), 1 h after operation (*P* = 0.005), similar pain score 2 h (*P* = 0.39), 3-4 h (*P* = 0.32), 6-8 h after operation (*P* = 0.05), then lower VAS 12 h after operation (*P* = 0.00006), and again similar VAS 1 day (*P* = 0.62) and 2 days after operation (*P* = 0.70). As for the VAS with movement, the SSNB group had higher pain score in PACU (*P* = 0.03), similar VAS 4-6 h after operation (*P* = 0.25), then lower pain score 8-12 h after operation (*P* = 0.01) and again similar VAS 1 day after operation (*P* = 0.3) compared with the ISB group. No significant difference was found for oral morphine equivalents use at 24 h (*P* = 0.35), duration of PACU stay (*P* = 0.65), the rate of patient satisfaction (*P* = 0.14) as well as the rate of vomiting (*P* = 0.56), and local tenderness (*P* = 0.87). However, the SSNB group had lower rate of block-related complications such as Horner syndrome (*P* < 0.0001), numb (*P* = 0.002), dyspnea (*P* = 0.04), and hoarseness (*P* = 0.04).

**Conclusion:**

Our high-level evidence established SSNB as an effective and safe analgesic technique and a clinically attractive alternative to interscalene block with the SSNB’S advantage of similar pain control, morphine use, and less nerve block-related complications during arthroscopic shoulder surgery, especially for severe chronic obstructive pulmonary disease, obstructive sleep apnea, and morbid obesity. Given our meta-analysis’s relevant possible biases, we required more adequately powered and better-designed RCT studies with long-term follow-up to reach a firmer conclusion.

## Background

Shoulder arthroscopic surgery can be associated with a 45% incidence of severe intraoperative and postoperative pain that can interfere with recovery and rehabilitation, which can be challenging to manage without large dose opioids. So, controlling postoperative pain while minimizing opioid administration is incredibly essential. Supplementing general anesthesia (GA) with a regional nerve block is recommended for reducing anesthesia’s intra-operative requirements, improving the quality of postoperative pain relief, easing postoperative rapid recovery [4, 5, 40].

Regional anesthetic techniques can control pain effectively, both at rest and on movement, allowing earlier mobilization without the adverse effects of opioids [36]. Among the various types of regional anesthetic techniques, the interscalene brachial plexus block (ISB) is a standard used nerve block technique for postoperative analgesia in patients undergoing shoulder surgery, as it has consistently been shown to significantly control postoperative pain, even in a small dose or single dose [2, 17, 18, 24, 26]. Moreover, ISB can be used to provide either surgical anesthesia or postoperative anesthesia. Many studies have demonstrated that ISB provides optimal analgesia in shoulder arthroscopic surgery in success rates of 87–100% [36]. Although it is useful for postoperative analgesia, ISB is challenging to perform, and it is associated with potentially serious complications such as diaphragmatic paresis from the phrenic nerve block, pneumothorax, brachial plexus injury, extended motor block inadvertent epidural anesthesia, and vertebral artery injection [15, 27]. Furthermore, there are relative contraindications in patients with severe chronic obstructive pulmonary disease because of phrenic nerve issues [15]. This clinical problem has recently received considerable attention, with several calls to seek alternatives to interscalene block in shoulder arthroscopy [9]. So, the need for a safer ISB alternative has prompted researchers to examine several options, including but not limited to the suprascapular block [14]. The suprascapular nerve block (SSNB) technique is a simple, easily reproducible technique that is thought to supply sensory fibers to approximately 70% of the shoulder joint and directly innervates the supraspinatus and infraspinatus muscles. The most common complication of SSNB is transient nerve palsy [6].We found few studies comparing the efficacy of SSNB and ISB for arthroscopic shoulder surgery. Some have found ISB to be superior [23, 39], whereas others have shown that SSNB provides noninferior analgesia [11, 25]. Our meta-analysis was to compare the analgesic efficacy of SSNB with ISB after shoulder arthroscopy.

## Methods

The current meta-analysis was registered on PROSPERO (International prospective register of systematic reviews) and the registration number was CRD42020205426.This meta-analysis was performed using a predetermined protocol following the PRISMA (Preferred Reporting Items for Systematic Reviews and Meta-Analyses) statement to assess the results’ quality to make sure our meta-analysis’s results reliable and veritable.

### Search strategy

SSNB and ISB during shoulder arthroscopy surgery. Web of Science, PubMed, Embase, Cochrane Controlled Trials Register, Cochrane Library, Highwire, CNKI, and Wanfang database were searched from 2010 through March 2021. The keywords used were “nerve block,” “regional,” “suprascapular,” “interscalene,” “shoulder,” “arthroscopic,” randomized controlled trials” in conjunction with Boolean operators “AND” or “OR.” We used Review Manager Software for MAC to perform the meta-analysis.

### Inclusion criteria

Studies were eligible if (1) the intervention was patients undergoing shoulder arthroscopic surgery with SSNB; (2) the comparator was patients undergoing shoulder arthroscopic surgery with ISB; (3) the study design was a randomized controlled trial (RCTs); and (4) the studies were required to contain at least one clinical outcome data. The exclusion criteria were as follows: (1) observational studies; (2) non-RCTs; and (3) studies with insufficient clinical outcome data.

### Data extraction process

Two reviewers (C.J.S and Q.M.) used a standardized form to extract data. A third reviewer (H.D.Y.) was used to resolve disagreements in eligibility, data extraction, or quality assessment. Extracted data included the primary data based on the following: first author, year of publication, participants, age, gender, body mass index, follow-up time, type of surgery, localization method, analgesia used in nerve block, analgesia used in PACU, and analgesia used in the ward. The included studies evaluated pain score with similar scores like VAS or NRS which is a line with anchor statements on the left (no pain) and on the right (extreme pain). Therefore, we equate NRS with VAS in statistical analysis. Because there were different kinds of opioid drugs in different studies and some are intravenous opioid drugs, some are oral opioid drugs. To reduce heterogeneity, we have converted various opioids drugs into the same morphine (oral). Conversions of amounts of different opioids used were performed using a web-based opioid conversion calculator (https://globalrph.com/medcalcs/opioid-conversions-calc-original-single-agent/).

### Assessment of studies

The studies’ methodological quality was assessed following the instructions in the Cochrane Handbook for Systematic Reviews of Interventions.

### Statistical analysis

RevMan software (version 5.3; The Cochrane Collaboration) was used for the analysis. We used a random-effects model for all analyses, as clinical heterogeneity was assumed to exist because of differences in standardization in anesthetic, nerve block techniques, diversity of shoulder surgeries performed, and the timing of assessment across studies. Data were summarized as the ratio of relative risk (rate of patient satisfaction, complications including the rate of subjective dyspnea, hoarseness, vomiting, local tenderness, Horner syndrome, and numb) or the difference between means (VAS at rest, VAS with movement, oral morphine equivalents use at 24 h and duration of PACU stay). Studies that did not report standard deviations (SDs) were calculated from *p* values, confidence intervals, or standard errors. The results were considered as a statistically significant difference when *P* values were less than 0.05.

## Results

**Fig. 1:**
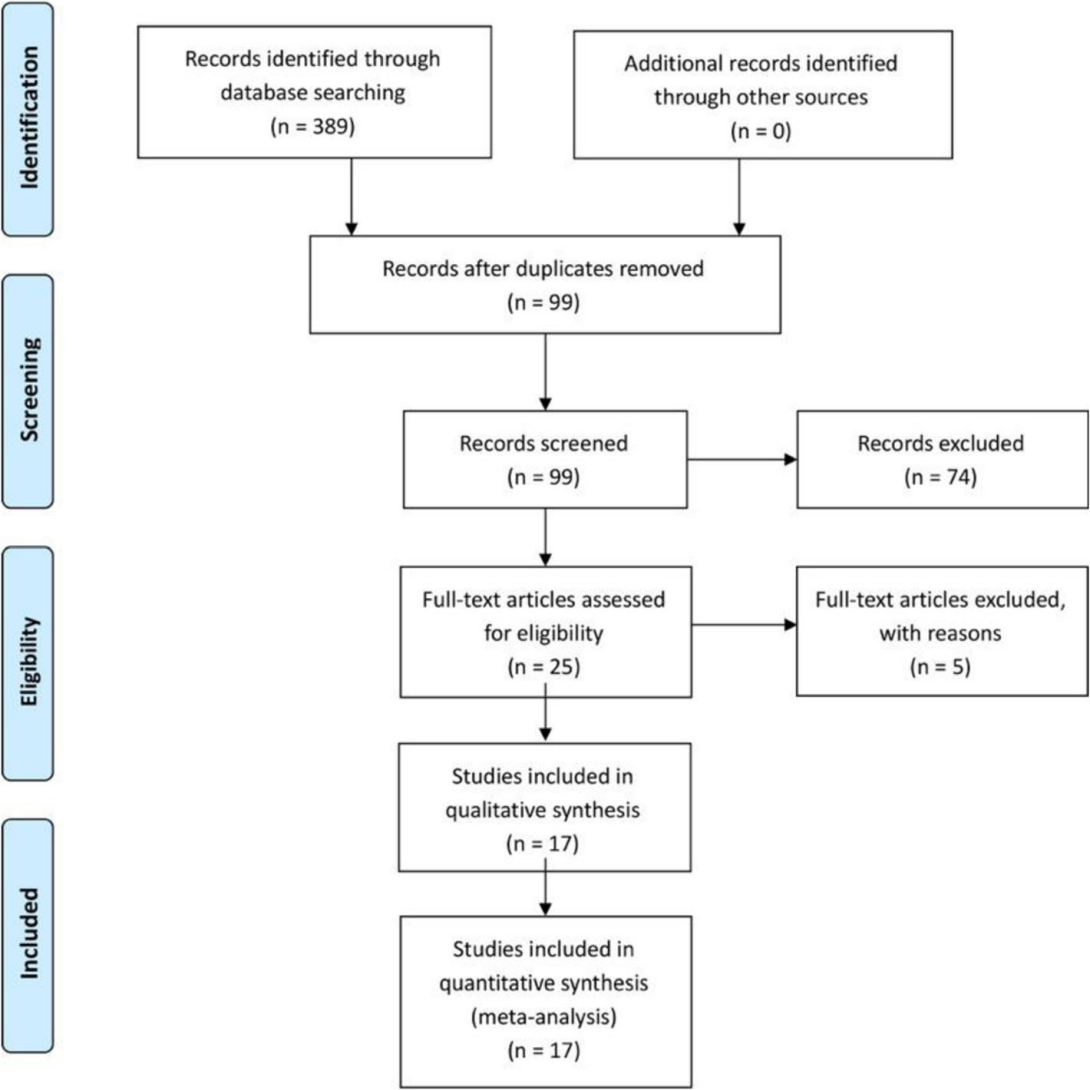
The search results and selection procedure. The literature search identified 389 citations. Of these, 290 duplicates were removed. After reviewing the 99 remaining articles’ titles and abstracts, we excluded 74 papers according to the inclusion and exclusion criteria; 25 full texts were retrieved. Because some articles did not compare the suprascapular nerve block with interscalene nerve block, five studies were excluded. Finally, we identified 1255 patients assessed in 17 randomized controlled trials

The literature search identified 389 citations. Of these, 290 duplicates were removed. After reviewing the 99 remaining articles’ titles and abstracts, we excluded 74 papers according to the inclusion and exclusion criteria; 25 full texts were retrieved. Because some articles did not compare the suprascapular nerve block with interscalene nerve block, five studies were excluded. Finally, we identified 1255 patients assessed in 17 randomized controlled trials [1, 3, 7, 11, 20, 21, 25, 28–31, 34, 35, 40, 42–44] (Fig. 1). Study baseline characteristics and general intervention information are summarized in Tables 1 and 2.

**Table 1:** Characteristics of included studies and patients

Study	Country	Sample sizes, n (SSNB/ISB)	Age, y, mean (SSNB/ISB)	% Female (SSNB/ISB)	BMI, kg/m^**2**^ (SSNB/ISB)	Follow-up	Surgery
**Abdallah 2020** **[**1**]**	Canada	67/69	46/40	NA	26.1/26.2	24 h	Shoulder arthroscopy
**Auyong 2018** **[**3**]**	USA	60/61	55/54	33/40	28.9/27.8	24 h	Arthroscopic rotator cuff or Bankart repair
**Cao 2019** **[**7**]**	China	25/25	57.72/56.8	40/60	24.69/24.38	48 h	Arthroscopic rotator cuff or Bankart repair
**Desroches 2016** **[**11**]**	France	28/25	60.8/56.5	39/36	NA	7 d	Arthroscopic rotator cuff repair
**Ikemoto 2010** **[**20**]**	Brazil.	15/15	57/54	73.3/66.7	NA	48 h	Arthroscopic rotator cuff repair
**Jiang 2017** **[**21**]**	China	24/23	56.4/55	62.5/65.2	23.2/22.9	24 h	Shoulder arthroscopy
**Kumara 2016** **[**25**]**	India	30/30	NA	NA	NA	24 h	Shoulder arthroscopy
**Li 2018** **[**28**]**	China	20/20	45.65/47.69	60/55	23.58/24	48 h	Shoulder arthroscopy
**Lim 2020** **[**29**]**	Singapore	20/20	40.3/42.8	25/35	27/25.3	24 h	Shoulder arthroscopy
**Liu 2020** **[**30**]**	China	54/53	NA	NA	NA	3 d	Shoulder arthroscopy
**Mai 2019** **[**31**]**	China	60/60	56.7/56.6	63.3/65	323.2/23.4	12 h	Shoulder arthroscopy
**Ovesen 2014** **[**34**]**	Denmark	23/22	48.95/48.70	70/50	NA	24 h	Arthroscopic subacromial decompression
**Petroff 2020** **[**35**]**	Germany	24/24	50/52	42/50	25.8/26	24 h	Shoulder arthroscopy
**Singelyn 2004** **[**40**]**	France	30/30	52/54	50/63	25.77/25.16	24 h	Arthroscopic acromioplasty
**Wang 2019** **[**42**]**	China	20/21	48.7/50.86	55/52.4	23.47/22.83	24 h	Shoulder arthroscopy
**Wiegel 2017** **[**43**]**	Germany	164/165	53/55	38		24 h	Shoulder arthroscopy
**Yao 2019** **[**44**]**	China	48/47	53,6/54.1	37.5/40.4	24.4/24.8	24 h	Arthroscopic rotator cuff repair

Abbreviations: *SSNB*, suprascapular nerve block; *ISB*, interscalene block

The detailed baseline characteristics information including country, number of participants, age, gender, BMI, follow-up time, and type of surgery

**Table 2:** Details of the nerve blocks and anesthesia used

Study	Localization method	Analgesia used in nerve block	Analgesia used in PACU	Analgesia used in ward
**Abdallah 2020** **[**1**]**	Ultrasound guided	Local anesthetic solution in 5-ml aliquots	NRS > 4, IV fentanyl in 25-μg increments every 5 min, as needed, up to a total of 100 μg, followed by IV morphine in 5-mg increments every 10 min up to a total of 20 mg or hydromorphone in 0.2-mg increments every 10 min up to a total of 3 mg.	Oral acetaminophen 300 mg plus codeine 30 mg combination tablets every 4 h, as needed, followed by oral oxycodone 5 to 10 mg every 4 h, as needed.
**Auyong 2018** **[**3**]**	Ultrasound guided	15 mL of 0.5% ropivacaine	NRS pain score of 4-6, 25 mg of fentanyl IV; NRS score 7-10, 50 mg of fentanyl IV.	NRS score 4-6, 5 mg of oral oxycodone; NRS score 7-10, 10 mg of oral oxycodone.
**Cao 2019** **[**7**]**	Arthroscopic guided (SNNB)/ Ultrasound guided (ISB)	20 mL of 0.2% ropivacaine	NA	100 mg of imrecoxib orally. If VAS > 3, 50 mg of flurbiprofen axetil IV; 6 h later, if VAS > 3, another 50 mg of flurbiprofen axetil IV; if VAS > 5, 50 mg of pethidine IM
**Desroches 2016** **[**11**]**	Ultrasound guided	20 mL of 0.75% ropivacaine.	1 g of acetaminophen, 100 mg of ketoprofen, 100 mg of tramadol IV. If VAS > 3, 3 mg of morphine IV; 5 min later, if VAS > 3, another 3 mg of morphine IV	Acetaminophen (325 mg, 6 times a day), ketoprofen (100 mg, twice a day), pantoprazole(20 mg, once a day), tramadol (37.5 mg, 6 times a day). If still in pain, oral morphine sulfate (10 mg, 6 times a day).
**Ikemoto 2010** **[**20**]**	Anatomic landmarks guided	2 mg/kg of 0.5% ropivacaine	NA	NA
**Jiang 2017** **[**21**]**	Ultrasound guided	20 mL of 0.375% ropivacaine+5mg of dexamethasone	If VAS > 4, 50 mg of pethidine IM	If VAS > 4, 50 mg of pethidine IM
**Kumara 2016** **[**25**]**	Electrophysiology-guided	20 mL of 0.5% bupivacaine with 75 mg of clonidine	If VAS> 4, 75 mg of diclofenac IM	If VAS> 4, 75 mg of diclofenac IM
**Li 2018** **[**28**]**	Ultrasound guided	20 mL of 0.375% ropivacaine	If VAS > 4, tramadol IV	If VAS > 4, tramadol IV
**Lim 2020** **[**29**]**	Ultrasound guided	15 ml of 0.5% ropivacaine	Intravenous morphine (up to 0.2 mg/kg)	Regular oral paracetamol 1 g every 6 h and etoricoxib 120 mg once daily
**Liu 2020** **[**30**]**	Ultrasound guided	6 mL of 0.3% ropivacaine	NA	NA
**Mai 2019** **[**31**]**	Ultrasound guided	20 mL of 0.375% ropivacaine+5 mg of dexamethasone	NA	NA
**Ovesen 2014** **[**34**]**	Anatomic landmarks guided	20 mL of bupivacaine (SSNB)/30 mL of ropivacaine (ISB)	NA	1 g of paracetamol 4 times a day, 600 mg of ibuprofen 3 times a day. If VAS > 3, patients received 3-5 mg nicomorphine hydrochloride IV followed by 5 mg ketobemidone.
**Petroff 2020** **[**35**]**	Ultrasound guided	10 ml of 1% ropivacaine	NA	Ibuprofen 600 mg up to four times or the synthetic opioid tilidine 100 mg up to twice in 24 h, as needed.
**Singelyn 2004** **[**40**]**	Anatomic landmarks guided	10 mL of 0.25% bupivacaine (SSNB)/20 mL of 0.25% bupivacaine (ISB)	If VAS > 3, 2 g of IV propacetamol, 30 min, if VAS > 3 5 or 10 mg of subcutaneous morphine	If VAS > 3, 2 g of IV propacetamol, 30 min, if VAS > 3 5 or 10 mg of subcutaneous morphine
**Wang 2019** **[**42**]**	Ultrasound guided	15 mL of 0.5% ropivacaine	If VAS > 3, tramadol IV	If VAS > 3, tramadol IV
**Wiegel 2017** **[**43**]**	Ultrasound guided	10 mL of 1% ropivacaine/20 mL of 0.75% ropivacaine	If NRS > 3, 3 mg of IV piritramide	If NRS > 3, 3 mg of IV piritramide
**Yao 2019** **[**44**]**	Ultrasound guided	15 mL of 0.5% ropivacane (SSNB)/20 mL of 0.5% ropivacaine (ISB)	If VAS > 3, 100 mg of tramadol IV, 10 min, if VAS > 3, 3 mg of morphine IV.	300 mg of oral ibuprofen 3 times a day, if VAS > 3, 100 mg of Tramadol IV; 10 min, if VAS >3, 2 mg of hydromorphone hydrochloride IM

Abbreviations: *SSNB*, suprascapular nerve block; *ISB*, interscalene block; *IV*, intravenous; *IM*, intramuscular; *NRS*, numerical rating scale; *VAS*, visual analog scale.; *PACU*, postanesthesia care unit; *US*, ultrasound

Details of the nerve blocks and anesthesia used including localization method, analgesia, used in nerve block, analgesia used in PACU and analgesia used in ward

**Fig. 2:**
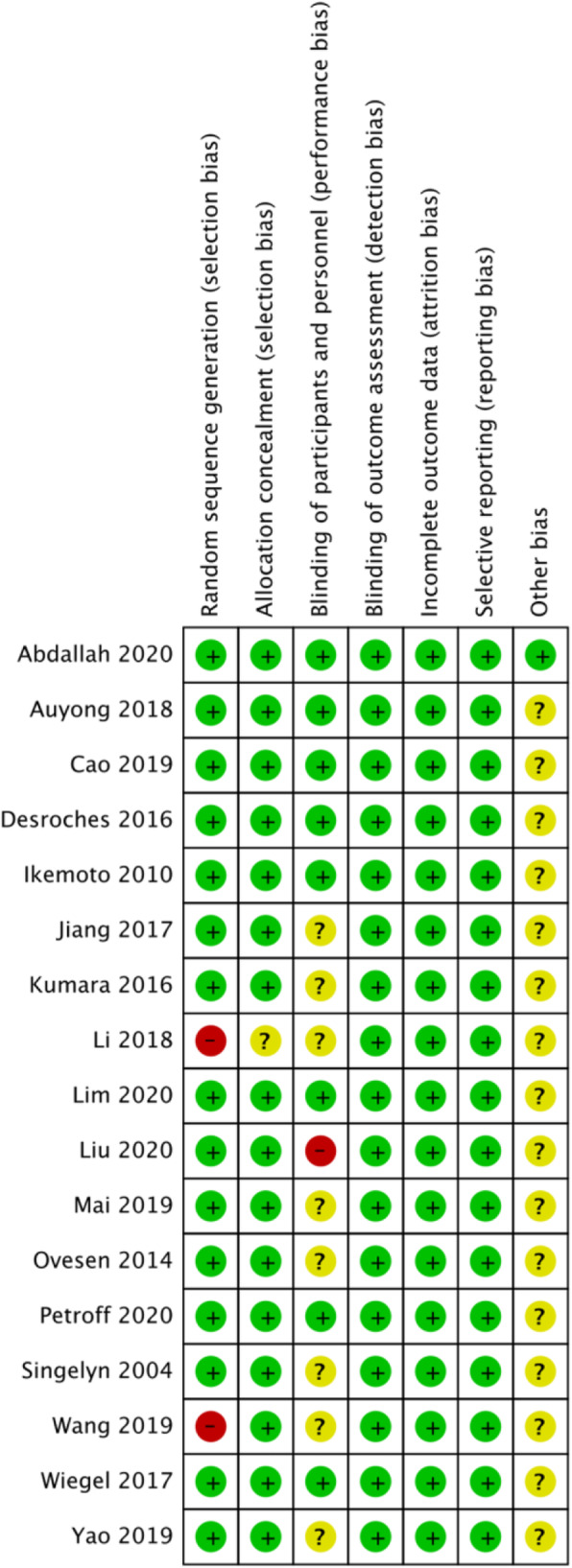
Risk of bias summary for included studies. +, no bias; −, bias; ?, bias unknown. Fifteen studies adequately described the correct randomization. Sixteen studies demonstrated sufficient allocation concealment. Eight studies described the blinding of participants and personnel. All seventeen articles described the blinding of outcome assessment, retained complete outcome data, and avoided selective reporting. We rated as unclear risk of other bias because we cannot ignore other potential dangers of biases

**Fig. 3:**
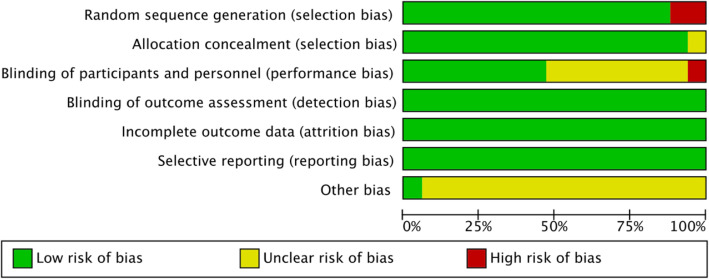
The risk of bias graph. The overall quality of the included studies was considered adequate

The risk of bias summary and bias graph for RCTs is shown in Figs. 2 and 3. Fifteen studies adequately described the correct randomization. Sixteen studies demonstrated sufficient allocation concealment. Eight studies described the blinding of participants and personnel. All seventeen articles described the blinding of outcome assessment, retained complete outcome data, and avoided selective reporting. We rated as unclear risk of other bias because we cannot ignore other potential dangers of biases. As a result, there is a low or moderate risk of bias in most of the articles reviewed.

### Outcome

#### VAS at rest

**Fig. 4:**
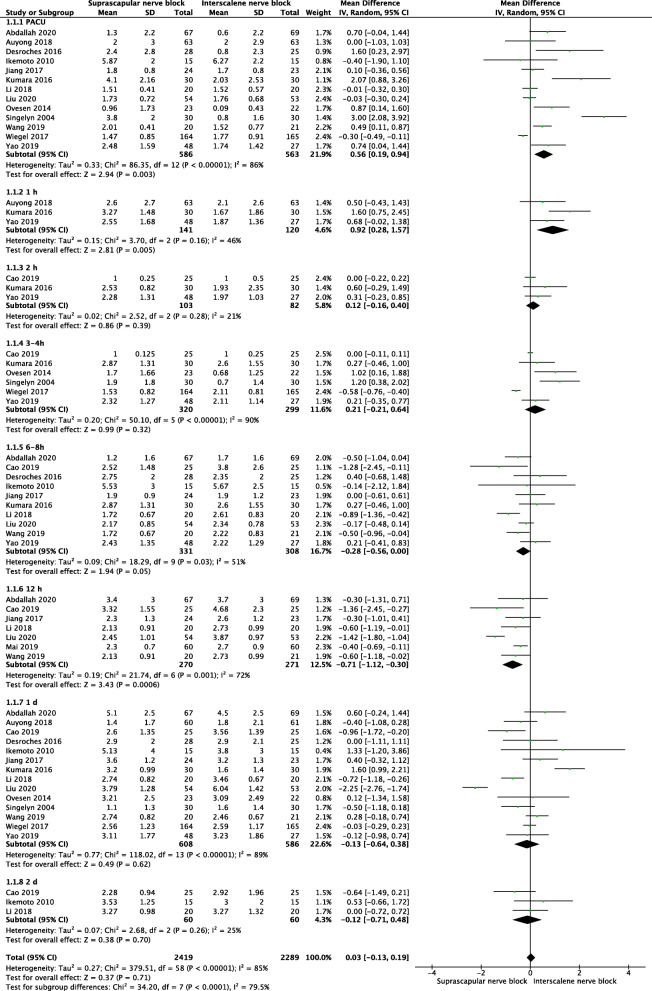
A forest plot diagram showing VAS at rest. The pooled results showed that SSNB group had higher VAS at rest in PACU (MD = 0.56, 95% CI [0.19, 0.94], *P* = 0.003) and 1 h after operation (MD = 0.92, 95% CI [0.28, 1.57], *P* = 0.005), however, lower VAS at 12 h after operation (MD = −0.71, 95% CI [−1.12, −0.3], *P* = 0.0006). No significant difference was found for VAS at rest 2 h after operation (MD = 0.12, 95% CI [−0.16,0.4], *P* = 0.39), 3-4 h (MD = 0.21, 95% CI [−0.21, 0.64], *P* = 0.32), 6-8 h (MD = −0.28, 95% CI [−0.56, 0.00], *P* = 0.05), 1 day (MD = −0.13, 95% CI [−0.64, 0.38], *P* = 0.62), and 2 days after operation (MD = −0.12, 95% CI [−0.71, 0.48], *P* = 0.7)

The pooled results showed that SSNB group had higher VAS at rest in PACU (MD = 0.56, 95% CI [0.19, 0.94], *P* = 0.003) and 1 h after operation (MD = 0.92, 95% CI [0.28, 1.57], *P* = 0.005), however, lower VAS at 12 h after operation (MD = −0.71, 95% CI [−1.12, −0.3], *P* = 0.0006). No significant difference was found for VAS at rest 2 h after operation (MD = 0.12, 95% CI [−0.16,0.4], *P* = 0.39), 3-4 h (MD = 0.21, 95% CI [−0.21, 0.64], *P* = 0.32 ), 6-8 h (MD = −0.28, 95% CI [−0.56, 0.00], *P* = 0.05), 1 day (MD = −0.13, 95% CI [−0.64, 0.38], *P* = 0.62), and 2 days after operation (MD = −0.12, 95% CI [−0.71, 0.48], *P* = 0.7) (Fig. 4).

#### VAS with movement

**Fig. 5:**
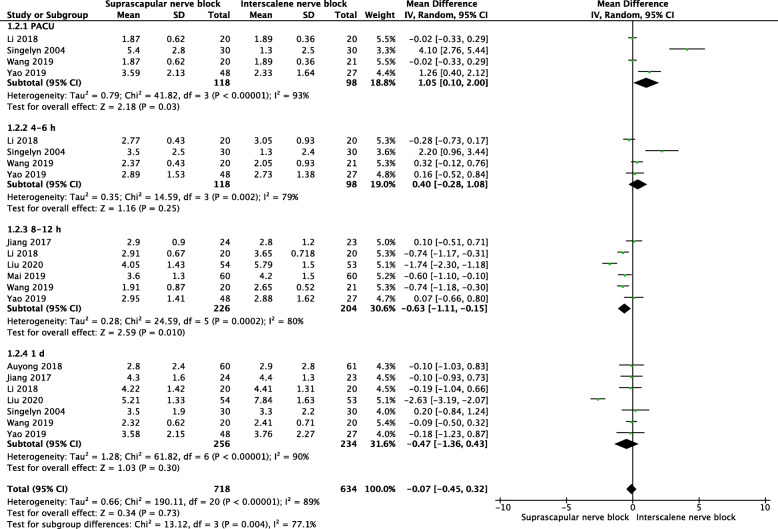
A forest plot diagram showing VAS with movement. The pooled results showed that SSNB group had higher VAS with movement in PACU (MD = 1.05, 95% CI [0.1, 2], *P* = 0.03), however, lower VAS 8-12 h after operation (MD = −0.63, 95% CI [−1.11, −0.15], *P* = 0.01). No significant difference was found for VAS with movement 4-6 h (MD = 0.4, 95% CI [−0.28, 1.08], *P* = 0.25) and 1 day after operation (MD −0.47, 95% CI [−1.36, 0.43], *P* = 0.3)

The pooled results showed that SSNB group had higher VAS with movement in PACU (MD = 1.05, 95% CI [0.1, 2], *P* = 0.03), however, lower VAS 8-12 h after operation (MD = −0.63, 95% CI [−1.11, −0.15], *P* = 0.01). No significant difference was found for VAS with movement 4-6 h (MD = 0.4, 95% CI [−0.28, 1.08], *P* = 0.25), and 1 day after operation (MD −0.47, 95% CI [−1.36, 0.43], *P* = 0.3) (Fig. 5).

#### Opioid drugs consumption

**Fig. 6:**
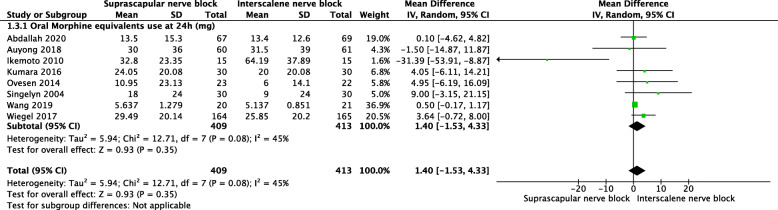
A forest plot diagram showing opioid drugs consumption. No significant difference was found for oral morphine equivalents use at 24 h (mg) (MD = 1.4, 95% CI [−1.53, 4.33], *P* = 0.35)

No significant difference was found for oral morphine equivalents use at 24 h (mg) (MD = 1.4, 95% CI [−1.53, 4.33], *P* = 0.35) (Fig. 6).

#### Nerve block-related complications

**Fig. 7:**
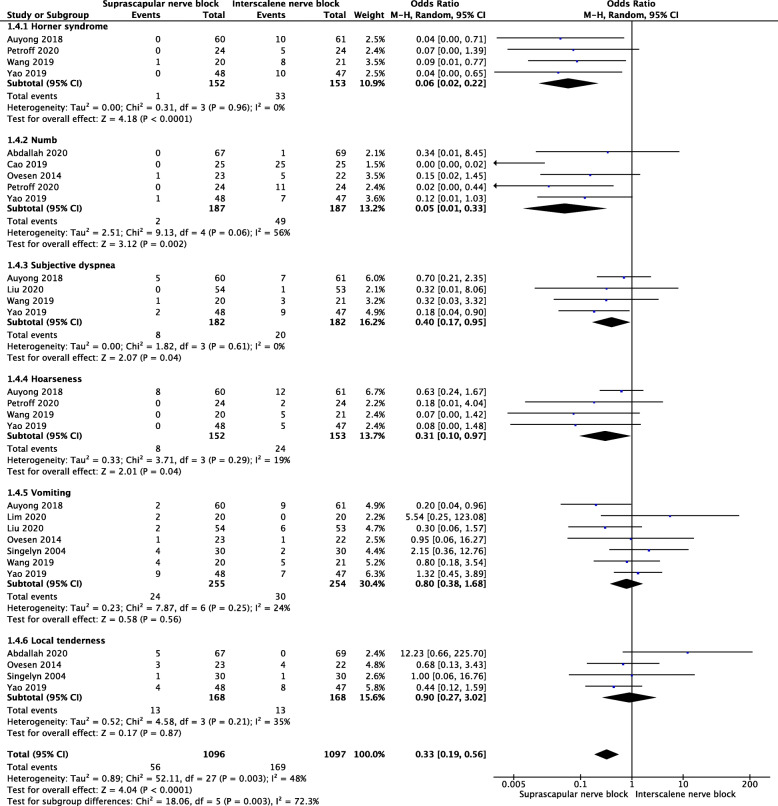
A forest plot diagram showing block-related complications. The pooled data showed that SSNB group had lower incidence of Horner syndrome (MD = 0.06, 95% CI [0.02, 0.22], *P* < 0.0001), numb (MD = 0.05, 95% CI [0.01, 0.33], *P* = 0.002), subjective dyspnea (MD = 0.4, 95% CI [0.17, 0.95]), hoarseness, (MD = 0.31, 95% CI [0.1, 0.97], *P* = 0.04). No significant difference was found for vomiting (MD = 0.8, 95% CI [0.38, 1.68], *P* = 0.56), local tenderness (MD = 0.9, 95% CI [0.27, 3.02], *P* = 0.87)

The pooled data showed that SSNB group had lower incidence of Horner syndrome (MD = 0.06, 95% CI [0.02, 0.22], *P* < 0.0001), numb (MD = 0.05, 95% CI [0.01, 0.33], *P* = 0.002), subjective dyspnea (MD = 0.4, 95% CI [0.17, 0.95]), hoarseness, (MD = 0.31, 95% CI [0.1, 0.97], *P* = 0.04). No significant difference was found for vomiting (MD = 0.8, 95% CI [0.38, 1.68], *P* = 0.56), local tenderness (MD = 0.9, 95% CI [0.27, 3.02], *P* = 0.87) (Fig. 7).

#### Duration of PACU stay

**Fig. 8:**
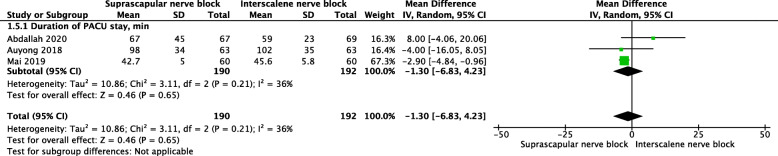
A forest plot diagram showing duration of PACU stay. No significant difference in the duration of PACU stay was found (MD = −1.3, 95% CI [−6.83, 4.23], *P* = 0.65)

No significant difference was found for the duration of PACU stay (MD = −1.3, 95% CI [−6.83, 4.23], *P* = 0.65) (Fig. 8).

#### Patient satisfaction

**Fig. 9:**
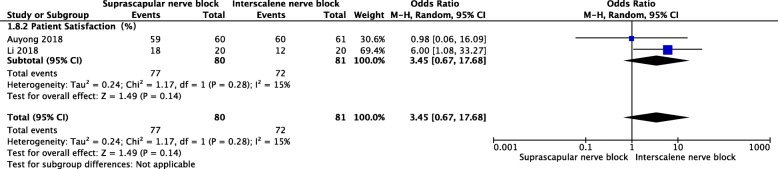
A forest plot diagram showing patient satisfaction. The pooled data showed that no significant difference was found for the rate of patient satisfaction (MD = 3.45, 95% CI [0.67, 17.68], *P* = 0.14)

The pooled data showed that no significant difference was found for the rate of patient satisfaction (MD = 3.45, 95% CI [0.67, 17.68], *P* = 0.14) (Fig. 9).

## Discussion

We only found two meta-analyses comparing SSNB with ISB. However, there is some difference between our study and the previous two meta-analyses. First, the inclusion standard is different. One meta-analysis by Kay [22] compared SSNB with ISB as well as anesthesia without a nerve block. Another meta-analysis by Hussain [19] included comparison of SSNB plus AXB and ISB. Second, the restriction of the previous meta-analysis to English language publications potentially limits the power obtained with the inclusion of non-English language studies. Third, the previous two studies did not separately evaluate the pain at rest and pain with movement. Under the current background of rapid recovery, patients need to move as soon as possible to achieve quick recovery, so it is essential to evaluate the pain with movement. Fourth, they did not analyze the duration of PACU stay and patient satisfaction. Thus, based on the current studies comparing SSNB with ISB during arthroscopic shoulder surgery, we only compare SSNB with ISB and include English language studies and Chinese RCTs. Moreover, our study added the analysis of pain at rest, pain with movement, duration of PACU stay, and patient satisfaction, which may provide a more exact conclusion and could be a supplement for the previous meta-analysis.

Our data of meta-analysis challenged the purported superiority of ISB over SSNB for shoulder surgery [12, 34, 39, 40]. Interestingly, the rebound phenomenon of increased pain in ISB group in our meta-analysis was not found in the previous two meta-analyses. The postoperative pain at rest and pain with movement at the individual time points suggested that ISB can provide better pain control that is limited to the PACU stay; however, there is a rebound phenomenon of increased pain in ISB group in the latter time. Compared with the ISB group, the SSNB group provides less pain control in the PACU stay, however, similar or superior pain control in the later time. There may be two reasons for the imperfect early pain control in the SSNB groups. First, the suprascapular nerve rarely has cutaneous innervation, and therefore the SSNB does not provide analgesia for the pain from skin incisions. Second, the suprascapular nerve supplies only 70% of the sensory fibers to the joint and capsule. The remaining 30% of the joint and capsule is innervated by the axillary, supraclavicular, subscapular, and pectoral nerves [8, 13] rather than the suprascapular nerve.

Management of shoulder surgery pain is often accomplished by using opioids; however, their use is often associated with side effects such as vomiting, nausea dysphoria, respiratory depression, and hormonal effects [32, 37]. The present high-level evidence suggested that the blocks are not different for the critical analgesic measures, namely, the difference between SSNB and ISB in the postoperative oral morphine consumption at 24 h. Likewise, the remaining analgesic outcome results, such as duration of PACU stay, patient satisfaction, and nerve block complications, including vomiting and local tenderness, were consistently not different between the two groups. In contrast, ISB was associated with a higher incidence of Horner syndrome, numb, subjective dyspnea, and hoarseness. From an anatomical perspective, the SSNB technique needs to perform blocks more distally along the brachial plexus, increasing the distance between block location and the phrenic nerve to decrease the phrenic nerve complications. Many studies also have shown that SSNB is a safe technique [19]. For example, the rate of minor complications was reported by only 0.6% (6/1005) [38]. Moreover, SSNB is an easy technique that can be performed using specific anatomic landmarks alone [33].

Though ISB provides superior pain control during the PACU stay, we did not find any significant difference between SNNB and ISB in terms of duration of PACU stay. Furthermore, no significant difference was found for patient satisfaction in SSNB and ISB, which may be explained by similar pain control in both nerve block techniques.

Our findings may have an impact on clinical practice. The minor analgesic advantages of ISB compared with the SSNB seem to be transient and limited to the immediate postoperative period (PACU stay). In contrast, the risk of block-related complications associated with ISB may outweigh its benefits in certain settings or patient populations, especially when SSNB can offer a safe and effective alternative in patients with severe chronic obstructive pulmonary disease [41], obstructive sleep apnea [10], and morbid obesity [16]. Our findings established the suprascapular block’s clinical benefits as an attractive, effective treatment for postoperative pain in patients undergoing shoulder surgery.

### Limitations

Our meta-analysis has limitations that should be acknowledged. First, heterogeneity was found across the included studies in terms of standardization in anesthetic, nerve block techniques, diversity of shoulder surgeries performed, and the timing of assessment, which precluded the pooling of many of outcomes. Second, some studies included in this review had smaller sample sizes, which may decrease the strength of their effect and limit external validity. Third, the number of the studies comparing the duration of PACU stay (three studies) and patient satisfaction (two studies) is too small. More adequately powered and better-designed RCT studies with these outcomes are needed to reach a firmer conclusion. Fourth, another bias inherent to the present study relates to the inability to blind the operator to the block technique being performed because of the interventions’ nature.

## Conclusion

ISB seems to offer minor analgesic advantages that are transient and limited to the immediate postoperative period. SSNB is equal or even better than ISB concerning postoperative pain control in the later time after operation. Furthermore, SSNB does appear to reduce the risk of Horner syndrome, numb, subjective dyspnea, and hoarseness. Our high-level evidence has established SSNB as an effective, safe, and clinically attractive alternative to ISB during arthroscopic shoulder surgery, especially for patients of severe chronic obstructive pulmonary disease, obstructive sleep apnea, and morbid obesity. Given our meta-analysis’s relevant possible biases, we required more adequately powered and better-designed RCT studies with long-term follow-up to reach a firmer conclusion.

## Data Availability

The datasets generated and analyzed during the current study are available from the corresponding author on reasonable request.

## Abbreviations

Cis: Confidence intervals
RCTs: Randomized controlled trials
RR: Risk ratio
OR: Odds ratio
VMD: Weighted mean difference
BMI: Body mass index
VAS: Visual analog scale
NRS: Numerical rating scale
SSNB: Suprascapular nerve block
ISB: Interscalene block
